# Monthly Summary

**Published:** 1882-12

**Authors:** 


### MONTHLY SUMMARY.

---

*The Speed of Thought*—We have several times given the readers of the *Journal* a report of what has been done by scientists to determine the rate at which nervous influence is transmitted through the telegraphic system of our bodies. Some recent investigations on the subject are thus summed up in the *American Journal of Arts and Sciences :*

Sensations are transmitted to the brain at a rapidity of about 180 ft. per second, or at one fifth the rate of sound ; and this is nearly the same in all individuals.

The brain requires one-tenth of a second to transmit its orders to the nerves which preside over voluntary motion; but this amount varies much in different individuals, and in the same individual at different times, according to the disposition or condition at the time, and is more regular, the more sustained the attention.

The time required to transmit an order to the muscles by the motor nerves is nearly the same as that required by the nerves of sensation to pass a sensation; moreover, it passes nearly one-hundredth of a second before the muscles are put in motion.

The whole operation requires one and one-fourth to two-tenths of a second. Consequently, when we speak of an active, ardent, mind, or of one that is slow, cold or apathetic. it is not a mere figure of rhetoric, but an absolute and certain fact that such a distinction, with varying graduations, really exists.

The method by which these nerve motions are measured is thus described :—

If a cylinder divided into 360° be caused to rotate 1000 times in a second, it is evident that the passage of one of those degrees before a given point is equal to the 1,360,000th part of a second; this may be divided by a microscope, so that a period of time equallying the ten millionth, or even the one hundred millionth, part of a second may be measured. By this arrangement it is possible to measure the rate of nervous impulse. Suppose an electric shock be given to the arm; it produces a sensation and a contraction of the muscles; then, by noting the interval of time between the shock and the contraction, of the muscles; the time occupied by the action of the brain to produce the contraction, however quick, will be ascertained. By trying this experiment on various parts of the body, the amount of sensibility of the different leading muscles maybe determined.—*Boston Journal of Chemistry.*

---

*The Use of Iodiform in the Dental Practice.*—It has been the aim of many thinking dentists to discover some remedy to save exposed pulps, in order to keep up the circulation and consequent nutrition of the tooth.

Thirty years ago Kœker was the first to recommend the saving of pulps when exposed, which has ever since been practiced with more or less success. Stimulated by the reports of Mosetig, in reference to the antiseptic and change-producing property of the iodiform, and encouraged by Underwood's article in regard to the power of iodiform to avert suppuration, etc., I concluded to give it a fair trial as to its virtue in the preservation of exposed nerves, and have, so far, been successful in nineteen cases of exposed pulps to save the teeth without destroying the nerve.

I cover the exposed nerve with a paste of pure iodiform and glycerine mixed in a small mortar, covering the paste with a pellet of cotton. and close the cavity with gutta-perch. taking care not to pack the latter so tight as to produce pressure upon the nerve. The further treatment, capping the filling of the tooth as well as the use of iodiform in the treatment of teeth where the nerve has been killed or dead, I will report in a dental journal some future time, and will only add here that I failed in three cases to save the tooth which was extracted at the request of the patient, after I made one application of the iodiform.

On microscopic examination of the nerve (longitudinal section) I found the whole field covered with new cells, showing an inflammatory condition of the organ.

The assertion that iodiform, like arsenious acid, is capable of destroying the vitality of the nerve is not confirmed, and has to be a priori precluded, when we consider in what large quantities this remedy is put upon and into wounds of all kinds, and how much mischief it would produce if such an assertion was correct.

---

*Excision of Superior Maxillary and Inferior Dental Nerves, for Neuralgia.*—"The fact that a new connecting medium sufficient to enable a nerve to perform its functions can be developed to the extent of an inch or more has been sufficiently attested. Any resection, therefore, which would embrace a less portion of nerve would in all probability be of only temporary benefit, unless the period of relief gained thereby would permit of such constitutional recuperation as would withstand any future attacks.

"My experience in the operation of resection for neuralgia has been extremely limited, but the success attending my efforts has been so satisfactory that I venture to offer it as an inducement for a more extended application of this method of relief"

A severe case of neuralgia coming under his care, it was decided to resect a portion of the inferior dental nerve. The disc of bone removed by the trephine failed to expose the nerve, and the patient bearing ether badly, it was decided to postpone further operative interference. Subsequently, both the superior maxillary and inferior dental nerves were excised.

The continuance of the pain for a few days after the operations and its return for a short time, seven months later, cannot well be accounted for. The clinical features of the case embraced all the symptoms described by Trosseau as characteristic of epileptiform neuralgia, which he believed to be an expression of true epilepsy, a view scarcely confirmed by the history narrated. Entire and permanent immunity from pain, return of appetite, ability to take solid food and comfortable sleep, were secured by the operations.—*Dr. W. Wallace in Pittsburg Med. Journal.*

---

*Tetanus from a Carious Tooth.*—A very remarkable case of fatal tetanus, ascribed to the irritation of a carious tooth, was reported some time back in one of the West of England journals. The patient was a shoemaker, residing at Bridgewater, who had enjoyed excellent health until he was seized with violent pain in the side of his head. He was treated in the first instance by a chemist for neuralgia, but the symptoms becoming aggravated Mr. Kemmis, a medical practitioner, was called in, He found the patient insensible, with his jaw locked and immovable. Treatment, however was unavailing; the man remained insensible, and died in a few hours. At the inquest Mr. Kemmis stated it as his opinion that death was due to tetanus brought about by a decayed tooth, and he characterized the case as a most extraordinary one—a statement with which every one will agree. Simple trismus from some form of dental irritation, generally the difficult eruption of wisdom teeth, is not a very rare phenomenon, and cases of it will be found recorded in several of our back numbers. But general and fatal tetanus from a similar cause is happily of extremely rare occurrence,

Mr. Tomes has recorded a case which was apparently due to the operation of pivoting, and Wedl has mentioned one in which tetanus followed the extraction of a tooth. In Mr. Tomes' case, as in the one the particulars of which are given above, death occurred very soon after the first appearance of muscular spasm.

---

*Therapeutics and Physiology.*—Dr. Geo. F Yeo concludes an address in the *Lancet* on "Experimental Physiology and Practical Medicine," as follows ;—

Rational therapeutics must grow out of physiological knowledge, as surely as a plant is the outgrowth of its roots. As the remote rootlets are the exact parts which are all important for the nutrition of the plant, so experiment feeds physiology, and thereby nourishes the art of medical practice. It would appear silly to ask to what rootlet any single fruit or flower on a widely-spreading tree owed its existence or nutrition, and so it is idle to expect that each, or even any, therapeutical agent or method of diagnosis should be traced to the definite experimental discoveries that may have led to its adoption or use. As the branches of our medical tree spread wider and wider, and its diagnostic flowers and therapeutic fruits become more numerous, we find that its physiological roots go deeper and deeper, in search of pabulum, and the experimental rootlets become still further removed from the more obviously useful and prolific part of the plant.

---

*Changes in the Secretion of Milk under Influence of Medicines.*—A few numbers ago the *Medical and Surgical Reporter* mentioned some experiments recently made to determine the influence certain medicines may or may not exert on the composition of the milk, when the drugs are given to the nursing mother. Dr. M. Stumpf has now instituted still more researches in this direction.

The investigations were mainly made upon a young goat, the feeding of which was carefully regulated. The influence of iodide of potash, ethylalcohol, lead, salicylic acid, morphia and pilocarpia were employed, and their effect noted concerning quality and quantity of milk: the transfer of these drugs to the milk was also specially examined into. The results were as follows :—

1. Iodide of potash diminishes, salicylic acid augments (probably) the quantity of milk ; alcohol, morphia and lead produce no change in the quantity of the milk, while pilocarpia does not enhance the secretion.

2. Salicylic acid augments the percentage of sugar in the milk, alcohol the fatty portion ; lead, morphia and pilocarpia leave the milk unchanged in this respect, while iodide of potash disturbs the function of the mammary gland and causes a change of all the parts of the milk.

3. Iodine, united to casein is transferred to the milk, but the quantity of the drug is subject to individual peculiarities, changing in different animals of the same species. In women the iodine disappears again in the milk as soon at its administration ceases ; in herbivorous animals the medicine is found for some time after cessation of introducing the drug into the system, still in the milk.

4. Alcohol is not transferred ; of lead, salicylic acid and the other remedies named, only traces were found.—*Med. and Surg. Reporter.*

---

*The Teeth in Diabetes.*—Dr. Magitot has after an examination of many diabetics, come to the following conclusions: First: Examination of the mouth of diabetics, furnishes a constant symptom of the disease. Second : This symptom is a lesion of the alveolar border which may be designated as an alveolar osteo-periostitis. Third : This manifestation appears at the outset of the disease, persists during its course and can, in consequence, be considered a pathognomonic symptom. Fourth : This alveolar affection. considered as a symptom of diabetes, presents three periods. Its first period is that of simple deviation of the teeth. Its second period is that of loosening of the teeth and alveolar catarrh. Each of these periods is in relation to the phase of the constitutional disease. The third period, that of the falling out of the teeth, corresponds to a more advanced state of glycosuria. Beside this last symptom they may occur, if the patient lives long enough, an osseous resorption which may, or may not, be consecutive to a gangrene of the gums. The appearance of this latter complication is evidence of a critical stage of the disease, as it ordinarily ushers in its fatal termination. The value, as a symptom, of the first stage of dental changes, remains to be determined. It must be obvious, however, that it can only occur in the more chronic forms of glycosuric diabetes.—*Exchange.*

---

*Preparations of Aconite*—Dr. Squibb, in his *Ephemeris*, writes concerning the value of the different preparations of aconite and its alkaloid. He concludes that the fluid extract of the root is the best preparation for internal use. The dose is one minim, which may be given every three hours, or oftener if needed. For external use, he says, there is probably no form better or more convenient than an oleate of aconita, made by dissolving two grains, or 130 milligrammes in 98 grains of oleic acid. A fluid ounce of oleic acid, weighing 412 grains, requires 8.25 grains of aconitia to make a 2 per cent. solution. Each minim of this oleate contains about 1.060 of a grain, and this quantity applied locally and repeated according to circumstances, should be an efficient dosage, and should in a short time produce constitutional effects by its absorption. It should be applied to the surface by the cork of the vial, or by some non-absorbent, without friction, and about the head and face it needs no covering; great care must be taken that it does not get into the eye. In using it around the eyes this caution must never be forgotten. If applied under the clothing it should be covered with oiled silk or rubber tissue. Local neuralgias are much better reached by the dermic or epidermic method of treatment. —*Med. Record.*

---

*Lancing the Gums of Children.*—It is very proper to lance the gums of children, when the gums are swollen, and either red from some inflammation, or *white* from pressure of a tooth *coming.*

The operator should *know* whether a tooth is pressing on the gum, and trying to make its way out. In this case *cut down to the new tooth,* until it is felt under the lancet. For incisors and cuspids, a straight line cut. For molars, a cross-cut.

How, *not* to do it: Not with a child sitting up, in your lap, or any one's lap.

*How* to do it: Let the operator and "nurse" sit close together, facing each other. The child is laid down, face upwards; the head in the operator's lap, the feet in the "nurse's" lap. The nurse holds the limbs of the child, quietly, so that it may not interfere.

With the left hand the operator takes the jaw between his fingers and slowly and firmly does the cutting.

There is no *false* cut, The child is still.—*Dr. Chase in Mo. Dent. Journal.*

---

*Toothless Persons*—Dr. Kilpatrick, of Navasota, Texas, thus writes to *Gaillard's Medical Journal*: "In your columns I notice the case of a man who died eighty years old, and who *never* had teeth. In corrobation of this I say I know a rough carpenter named Richardson, living in Catahoula Parish, La., who never had teeth. He was a dark skinned man of bilious temperament, very seldom sick, and at that time, 1854–60, was about fifty years old. I also know a lady here in this town who had never had teeth. She was from South Carolina. She was of ordinary size, fair complection, nervous temperament, intelligent, parents wealthy, married and the mother of three children. Her parents were near relations, and theirs were also, and I attributed the oddity to that. She wore artificial teeth after being grown. She also married her cousin. She has been dead about twelve years.

---

*Varnish Your Cavities*—Dr. Chase in the *Mo. Dent. Journal* says:

Cavities to be filled with either gold or other metals, deserve to be varnished. It seals up the open tuble. It covers the dental surface with a non conducting material. It cuts off electrical action between the dentine and filling. It in a great measure prevents chemical action caused by leaky plugs. It prevents oxides of metals from penetrating and staining dentos.

Dry the cavity thoroughly, then wipe it with sandarach varnish. Wipe it again nearly dry, and put in the filling. I say *nearly* dry, for the water present in the dentos will draw the alcohol from the varnish and leave only a dry gum on the surface of the cavity walls.

I speak from an experience of many years and am sure that this varnishing should never be omitted.

---

*To Gild Without a Battery.*—Small articles like rings, buttons, etc,, may be gilded as follows: Digest a small fragment of gold with about ten times its weight of mercury until it is dissolved, shake the amalgam together in a bottle, and after cleansing the articles coat them uniformly with the amalgam. Then expose them on an iron tray heated to low redness for a few minutes—the mercury volatizes, leaving the gold attached as a thin coating to the articles. The heating should be done in a stove, so that the poisonous mercurial fumes may pass up the chimney.—*Scientific American.*

---

*Excision of the Tongue.*—Mr. Croly, of Dublin, performs the operation for cancer of the tongue, even when the disease is situated in the anterior portion of the organ. His first ligatures each lingual artery close to the hyoid bone, through a curved incision, reaching from the symphysis down to the hyoid bone. and up and back to the angle of the jaw. Through these incisions he withdraws the tongue, as in Regnoli's operation, and removes the requisite amount of it by the benzoline cautery. Lastly, he divides the gustatory nerve where it lies along the inner border of the jawbone.

---

*Swallowing Artificial Teeth.*—The London *Lancet* reports a ase of swallowing artificial teeth by a man subject to epileptic fits. A partial denture was swallowed, and expelled *per rectum* twenty-four hours afterwards. The patient, after cleaning the plate, placed it *in situ*, and all was was well for nine years when the same plate took another visit through the pylorus, but was thirty-one days *en route* this time.

